# Preparation of Colorimetric Sensor Array System to Evaluate the Effects of Alginate Edible Coating on Boiled-Dried Anchovy

**DOI:** 10.3390/foods12030638

**Published:** 2023-02-02

**Authors:** Byungchan Cho, Korakot Charoensri, Hansol Doh, Hyun jin Park

**Affiliations:** 1 Department of Biotechnology, College of Life Sciences and Biotechnology, Korea University, 145 Anam-ro, Seongbuk-gu, Seoul 02841, Republic of Korea; 2 Department of Food Science and Biotechnology, Ewha Womans University, 52 Ewhayeodae-gil, Seodaemun-gu, Seoul 03760, Republic of Korea

**Keywords:** colorimetric sensor array, food quality, boiled-dried anchovy, edible coating, alginate

## Abstract

The colorimetric sensor array (CSA) is a simple, rapid, and cost-effective system widely used in food science to assess food quality by identifying undesirable volatile organic compounds. As a prospective alternative to conventional techniques such as total volatile basic nitrogen, peroxide value, and thiobarbituric acid reactive substance analysis, the CSA system has garnered significant attention. This study evaluated the quality of edible-coated food products using both conventional and CSA methods in order to demonstrate that the CSA approach is a feasible alternative to conventional methods. Boiled-dried anchovies (BDA) were selected as the model food product, and the sample’s quality was assessed as a function of storage temperature and incubation period using conventional techniques and the CSA system. The surface of BDA was coated with an edible alginate film to form the surface-modified food product. The conventional methods revealed that an increase in storage temperature and incubation time accelerated the lipid oxidation process, with the uncoated BDA undergoing lipid oxidation at a faster rate than the coated BDA. Utilizing multivariate statistical analysis, the CSA approach essentially yielded the same results. In addition, the partial least square regression technique revealed a strong correlation between the CSA system and conventional methods, indicating that the CSA system may be a feasible alternative to existing methods for evaluating the quality of food products with surface modifications.

## 1. Introduction

The colorimetric sensor array (CSA) may serve as a promising analytical technique for evaluating food quality owing to its relatively high sensitivity, simplicity, and time- and cost-effectiveness. Dyes, which are used to detect diverse analytes in the CSA system, may be advantageous as they act akin to a mammalian olfactory system [1]. As the CSA technique is based on dye–analyte interactions, including van der Waals forces, hydrogen bonding, dipolar and acid–base interactions, charge transfer, π-π complexation, and physical adsorption [2], its analytical ability is better than that of the electronic nose, which can be interrupted by the surrounding atmosphere [3]. Moreover, the CSA technique does not require a complicated pretreatment process, which is essential for conventional food quality detection methods [4,5]. As a result, CSA systems have been used widely to determine food quality and detect volatile toxic compounds in recent years [6]. For instance, the CSA system has been used to evaluate the quality of water [7], fish products [5], chicken breast [4], coffee [8], beer [9], and soft drinks [10].

Anchovy is one of the most popular marine products in Northeast Asia [11]. Earlier reports have described that anchovy helps reduce blood cholesterol levels and maintain normal blood pressure with the help of n-3 polyunsaturated fatty acids (PUFAs) [12,13]. However, the high levels of PUFAs in anchovies can cause rapid lipid oxidation in a short incubation period that can lead to adverse effects, such as undesirable off-flavor [14]. Accordingly, raw anchovies are usually salt-fermented, boiled, and dried to circumvent the adverse effects, as well as extend their short shelf-life [15]. Nevertheless, they are still subject to quality degradation due to lipid oxidation. Therefore, preventing lipid oxidation from the surrounding environmental conditions in anchovies assumes high significance in the fish industry.

An edible coating is one of the surface modification methods of food products for extending their shelf-life. This strategy was used in this study to improve the quality and freshness of anchovies through the formation of a protective barrier against moisture, carbon dioxide, respiration, and oxygen, which are considered critical factors in the lipid oxidation of fish products [11,16]. Alginate, a polysaccharide derived from brown algae, is composed of a varying sequence of (1 → 4)-linked α-L-guluronic (G) and β-D-mannuronic (M) residues [17]. Since it is widely used in the biomedical, food, cosmetic, and pharmaceutical industries due to its biocompatibility and unique colloidal properties that can form a strong film and gel with calcium ions [11,16,18,19,20], it was used in this study as an edible coating agent on the surface of boiled-dried anchovy (BDA). Several previous studies reported the use of alginate edible coating with various food products, including *Merluccius* spp. fillets [21], strawberries [22], pansies [23], fresh northern snakehead fish fillets [24], and refrigerated bream [25].

Based on the above study results, our research team expected that the alginate edible coating could similarly prevent rapid lipid oxidation in BDA as well. However, to the best of our knowledge, since no study has evaluated the quality of fish products after edible coating using the CSA system, these results were compared to those achieved with conventional methods to determine whether the CSA system works for surface-modified food products. In this study, based on the volatile organic compounds produced by the degradation of anchovy, especially carbonyl compounds, all dyes (pH indicators) were selected with several related studies of colorimetric sensor arrays [4,5,8,26,27,28] and the results of total volatile basic nitrogen (TVB-N), peroxide value (POV), and thiobarbituric acid reactive substances (TBARS) were evaluated for verification, which is considered as the conventional means for evaluation of lipid oxidation of fish products [5,23,24,25,26]. Therefore, the aims of this study were (1) to evaluate the quality and freshness of edible-coated BDA using the CSA system and (2) to compare the results of CSA methods to those obtained with conventional methods (TBARS, TVB-N, and POV tests) to determine whether the CSA system prepared and used in this study has potential for practical application.

## 2. Material and Methods

### 2.1. Materials

BDA was purchased from Wan-do Kumil-Suhyup (Wan-do, Jeollanam-do, Republic of Korea). Nine pH indicators (thymol blue, bromocresol green, cresol red, bromocresol purple, neutral red, bromoxylenol blue, alizarin, methyl red, and metanil yellow) were purchased from Sigma Aldrich Chemical Co., Ltd. (St. Louis, MO, USA). Ethanol (99.9%) was obtained from Duksan Pure Chemicals (Ansan-si, Gyeonggi-do, Korea). Polyvinylidene fluoride (PVDF) membranes were sourced from Merck KGaA (Darmstadt, Hesse, Germany). Sodium alginate, glycerol, calcium chloride, magnesium oxide, glacial acetic acid, chloroform, sodium thiosulfate, potassium iodide, trichloroacetic acid (TCA), butylated hydroxytoluene (BHT), and thiobarbituric acid (TBA) were purchased from Duksan Science (Ansan, Gyeonggi, Republic of Korea).

### 2.2. Edible Coating on the Surface of BDA

A sodium alginate solution (1% *w*/*v*, dissolved in deionized (DI) water) was prepared at 70 °C. Glycerol, used as a plasticizer, was added to the prepared sodium alginate solution at a 20% (*w*/*w*) ratio (based on the alginate mass content). The prepared sodium alginate solution was cooled down to 25 °C, and the BDA samples were coated with this solution using the dip-coating method. Each BDA was dipped in the sodium alginate solution for 1 min and then air-dried until the moisture content reached approximately 15%. Afterwards, the coated BDA sample was immersed in a 2% (*w*/*v*, dissolved in DI water) calcium chloride solution for 1 min to form cross-linking, and air-dried again until the moisture content reached about 15%. The coated BDA sample was incubated for 30 days at three different storage temperatures (25, 35, and 45 °C) (Supplementary Materials Table S1).

### 2.3. Quality Verification of BDA with Conventional Methods

#### 2.3.1. Determination of TVB-N Content

The TVB-N content of BDA was determined using the steam distillation technique [27]. Briefly, the BDA sample (5 g) was homogenized with 50 mL of DI water. After magnesium oxide (2 g) was added, the mixed solution was heated at 80 °C for 15 min, and the evaporated solution was collected in a round-bottom flask containing 25 mL of 2% (*w*/*v*, dissolved in DI water) boric acid and methyl red. Then, the obtained solution was titrated with 0.1 N of sulfuric acid. The titration point was identified when the green-colored solution turned pink. The TVB-N value is expressed in units of mg/100 g.

#### 2.3.2. Determination of POV

The POV was determined using the American Oil Chemists’ Society (AOCS) technique Cd 8-5. (AOCS, 1998). Briefly, the BDA sample (5 g) was extracted with 20 mL of a mixed solution (acetic acid: chloroform (3:2, *v*/*v*)) and 0.5 mL of saturated potassium iodide solution for 1 min. Thereafter, an excess amount of starch solution (1 mL, 3%, *w*/*v*, dissolved in DI water) was added as an indicator. After vigorous stirring, the mixture was titrated with 0.1 M of sodium thiosulfate solution. The color changed from yellow to transparent under constant stirring conditions. The volume of sodium thiosulfate was recorded to calculate the POV, expressed in units of meq/kg.

#### 2.3.3. Determination of TBARS

Briefly, BDA (5 g) was homogenized at 260 rpm for 1 min using a stomacher (Stomacher 400 circulator, Seward Laboratory System Inc., FL, USA) with 25 mL of 7.5% (*w*/*v*, dissolved in DI water) TCA solution and 1 mL of 0.5% (*w*/*v*) butylated hydroxytoluene in hexane. The mixture was subsequently centrifuged at 3134× *g* for 30 min at 25 °C, and the residue was filtered with filter paper (Whatman grade 1, qualitative filter paper, pore size: 11 μm). The filtered solution (5 mL) was then mixed with 0.02 M of TBA solution (5 mL) and heated at 70 °C for 30 min. After the reaction, the mixture was cooled on an ice bath and the absorbance was measured at 532 nm using a UV-VIS spectrophotometer (Optizen POP, Seoul, Republic of Korea). TBARS is expressed as mg malondialdehyde (MDA)/kg sample.

### 2.4. Quality Verification of BDA Using the CSA System

Nine pH indicators (5 mg) were dissolved in 10 mL of ethanol (99.9%). These stock solutions were then degassed for 10 min in a water bath sonicator. After degassing, 5 μL of the stock solution was placed on PVDF membrane sensor arrays of 3 × 3 formats (3 × 3 cm^2^, width and height, respectively) to prepare the dye for the CSA system. Then, the arrays were dried in a fume chamber for 30 min before analysis in the CSA system. Details of the dyes on the arrays are presented in Table 1.

**Figure 1:**
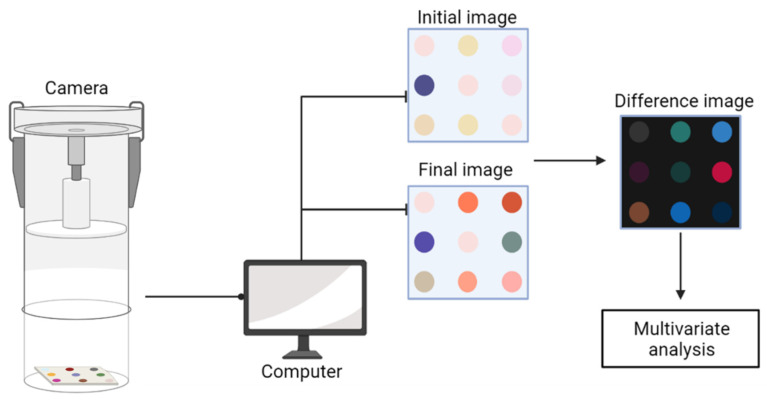
Schematic diagram of color sensor array system.

The prepared BDA samples (5 g) were placed in 90 mm polystyrene Petri dishes for CSA analysis. Initially, the sensor array was attached to the inside of the Petri dish lid and incubated for a minute. After the reaction, the sensor arrays were removed immediately from the lid before the analysis. Thereafter, sensor arrays were placed in the monitoring system and imaged using a camera. The recorded digital RGB values of the sensor arrays were obtained with the CSA software system. All samples were tested in triplicate under the same conditions. The process of the CSA system is depicted in Figure 1. Images indicating the color changes of the nine pH indicators in the array induced by the volatile carbonyl compound from the degradation of BDA were recorded and changes of dyes were captured using the color analysis software of an online monitoring system. After this, color analysis software was used to obtain the color change values of RGB (red, green, blue) by subtracting the initial RGB value from the final RGB value. To determine the color changes of BDA, the arrays consisting of nine dyes with a total vector dimension of 27 were utilized. The differences between color changes in an image are equivalent to the differences between the three N-dimensional vectors, where N is the number of dyes. Additional statistical analyses were performed using the acquired data from the CSA system. Abbreviations in this study are described as “x(N)C BDA day y,” where “x” indicates the storage temperature, “(N)C” means “(non)-coating”, and “y” represents the storage day(s).

### 2.5. Statistical Analysis

All data are presented as mean ± standard deviation. Statistical significance was determined using one-way analysis of variance (ANOVA) on IBM SPSS version 25, followed by ANOVA with Duncan’s test (*p* < 0.05). Multivariate statistical methods were performed with linear discriminant analysis (LDA) and partial least square regression (PLSR). The PLSR method was implemented using a calibration set, a prediction set accompanied by a correlation coefficient ($r_{c}{}^{2},\;r_{p}{}^{2})$, and root mean square error (RMSEC and RMSEP). (Note: $r_{c}{}^{2}$: calibration set of correlation coefficient, $r_{p}{}^{2}$: prediction set of correlation coefficient, RMSEC: calibration set of root mean square error, and RMSEP: prediction set of root mean square error.)

These indices were calculated using the following equations:

(1) $$r^{2}=1-\frac{\sum_{i=1}^{m}{\left(y_{i}-{\hat{y}}_{i}\right)}^{2}}{\sum_{i=1}^{m}{\left(y_{i}-\bar{y}\right)}^{2}}$$

(2) $$\mathrm{RMSE}\;=\;\sqrt{\frac{\sum_{i=1}^{m}{\left({\hat{y}}_{i}-y_{i}\right)}^{2}}{m}}$$

where ***m*** is the number of variables, $y_{i}$ and ${\hat{y}}_{i}$ are the estimated and predicted values of $i_{th}$ observation, respectively, and $\bar{y}$ is the mean value of the estimated values. The PLSR prediction sets were carried out by comparing the values of TVB-N, POV, TBA, and the CSA sensor dyes with 27 color vectors. A total of 90 variables were divided into two sets for application in the prediction model. One variable was selected from all three variables. Among the selected data, 60 variables were used for the prediction model, and 30 variables were used for the calibration model.

## 3. Results and Discussion

### 3.1. Quality Verification Using Conventional Methods

The TVB-N level is considered an indicator of food quality and freshness of fish products because it is used to estimate the quantity of protein hydrolysis intermediates that are produced in the mechanism of lipid oxidation in fish products [5,25,29,30,31,32]. The coated BDA samples showed lower TVB-N values than the non-coated BDA samples. In addition, after 20 days of incubation at 25 and 35 °C, non-coated BDA samples had higher TVB-N values than the acceptable range (<25 mg/100 g), while those of coated BDA samples were in the acceptable range [27,33]. The non-coated BDA samples stored at 45 °C had higher values than the acceptable TVB-N range after 10 days of incubation, whereas coated BDA retained values within the acceptable range. However, after 20 days of storage at 45 °C, both coated and non-coated BDA samples did not show acceptable TVB-N values. This result may be due to the accelerated rate of protein decomposition induced by high temperatures during the storage period. Overall, the protein decomposition rate was delayed by the alginate edible coating [25,33].

**Figure 2:**
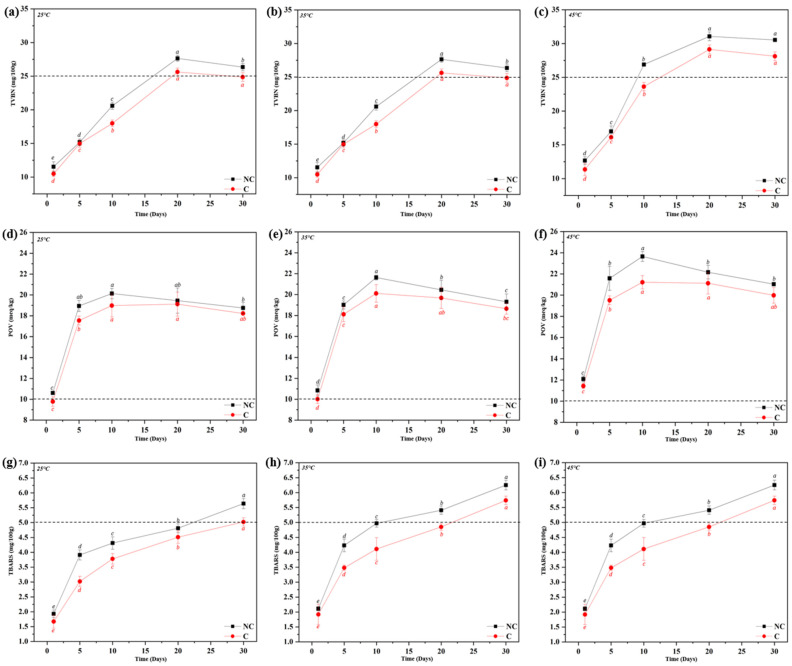
The levels of TVBN (**a**–**c**), POV (**d**–**f**), and TBARS (**g**–**i**) of BDA during storage period at 25, 35, and 45 °C, respectively. (Black dotted line indicates the acceptable range of the levels of TVBN, POV, and TBARS value). (Note: Different italic letters (*a*–*e*) show significantly different values (*p* < 0.05) according to Duncan’s multiple range tests.)

The POV test measures the amount of hydroperoxide, which is one of the major intermediates of lipid oxidation and the indicator of oxidative rancidity [34]. The POV results for BDA samples are illustrated in Figure 2d–f. The acceptable range of POV in fish products is considered approximately 10 meq O_2_/kg of fish product [35]. Overall, most of the BDA samples exceeded the acceptable range in all conditions, except the coated samples that were stored at 25 and 35 °C for 1 day. The POV in all samples gradually increased over the five days. The increased temperature accelerated the oxidation rate of both the coated and non-coated BDA samples. For instance, the BDA samples stored at 45 °C had a higher POV than those stored at other temperature conditions for the same incubation period. A similar trend was reported in previous studies [15,36]. The POV in all conditions increased during the ten days of incubation and slightly decreased by the end of the storage period because no further peroxide could be produced after ten days. However, the coated BDA samples had relatively lower POV than the non-coated BDA samples in all cases, which indicates that the alginate edible coating can delay severe lipid oxidation.

The TBARS test aims to quantify MDA, one of the final products in lipid oxidation. The changes in TBARS levels during the incubation period for all BDA treatments are shown in Figure 2g–i. The TBARS values increased in all cases over the storage period, regardless of the presence of coating. However, the increased TBARS values in coated BDA were less than those of non-coated BDA in all storage conditions. In addition, non-coated BDA had a TBARS value over the acceptable range (approximately 5 mg MDA/kg for fish products) after 20 days of incubation at 35 and 45 °C [37]. These findings suggest that temperature is the main reason for lipid oxidation, and the alginate edible coating system can delay the rate of lipid oxidation. Similar observations were made for coated meat, where plant-based hydrocolloid edible coating could effectively postpone lipid oxidation [38,39,40].

Overall, the TVB-N, POV, and TBARS tests revealed that lipid oxidation is promoted primarily by high temperatures during the incubation period. As expected, the alginate edible coating system could delay the lipid oxidation process of BDA because the coating layer acted as a barrier against the surrounding environment during storage [41].

### 3.2. Quality Verification Using the CSA System

#### 3.2.1. CSA Response

**Figure 3:**
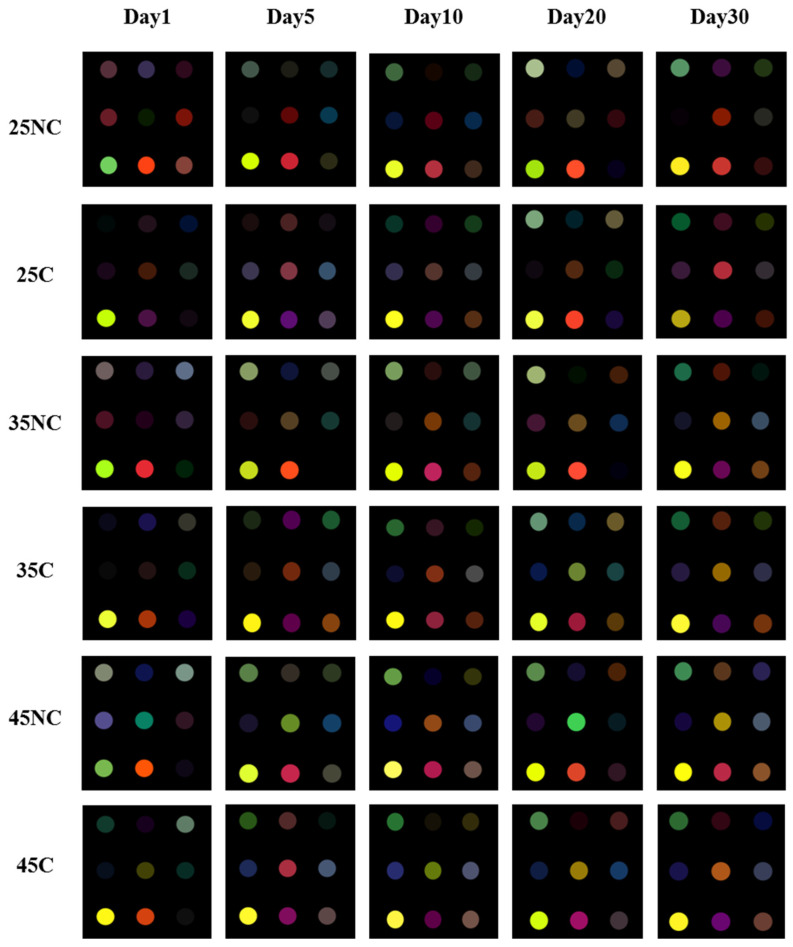
Different images of CSA of boiled-dried anchovy during storage period.

The lipid oxidation in fish products is the reason for undesirable flavor. It results from carbonyl compounds such as aldehydes and ketones. This offensive flavor is frequently caused by the lipoxygenase-mediated reaction of fish products that have high levels of polyunsaturated fatty acids. The final products of lipid oxidation in anchovies, such as volatile aldehydes with 5–10 carbons and ketones (3,5-octadiene-2-one and/or 2-undecanone), are the reason for the undesirable flavor [11]. The dyes used in this study for the CSA system were specifically designed to detect these volatile carbonyl compounds. In our CSA system, each VOC produced from BDA during lipid oxidation reacted with the dyes, and these images are shown in Figure 3.

From Table 1, three dyes indicating blue, yellow, and red color (No. 1, 5, and 8) exhibited significant color changes, and these were selected as the main dyes for further analysis. It was observed that the color differences between the samples stored for the same incubation period were significantly different for the main target dyes. In general, three factors determined the color differences in this study: the presence of edible coating, the storage temperature, and the incubation period. In the case of the non-coated BDA samples, the color changed more rapidly than in the coated BDA samples during the entire incubation period. This is because the coated BDA samples showed relatively slower lipid oxidation than the non-coated BDA samples owing to the edible coating. In addition, samples stored at higher temperatures and for a longer period of incubation exhibited higher oxidation levels than those stored at lower temperatures.

The dye labeled No. 1 displayed a green color that increased in brightness as oxidation progressed. The dye exhibited a green color with higher brightness for non-coated BDA samples than for coated BDA samples stored for the same incubation period. In non-coated BDA, the dye labeled No. 1 responded after the first day of incubation at all temperatures. On the other hand, there were nearly no responses after the first day of storage at 25 and 35 °C and only a light response at 45 °C in the case of the coated BDA samples. In the case of dye No. 5, the color of the dye changed to red, yellow, and green as the oxidation proceeded. Significant color changes were observed in non-coated BDA samples stored at 25 °C on day 5. However, coated BDA exhibited a red color after 30 days of storage at 25 °C owing to the presence of coating. Moreover, in non-coated samples stored at 35 °C, the red color appeared on day 10 and the yellow color on day 20, but no green color was observed throughout the study period. In contrast, coated BDA samples stored at 35 °C showed a red color on days 5 and 10. When the storage temperature increased to 45 °C, the green color was observed in the non-coated BDA samples from the first day of storage, whereas a red color appeared in the coated samples after 5 days of storage. The green color may have appeared between days 10 and 20. Additionally, the color of dye No. 8 changed to red as the oxidation proceeded.

In non-coated BDA, the color of dye No. 8 changed to red, while a purple color was observed in coated BDA, indicating that the dye was less affected by oxidation (except for the coated samples stored at 25 °C on day 20). At the same storage temperature and period, it was found that the redness of non-coated BDA samples was always higher than that of coated BDA samples.

Overall, it can be concluded that the lipid oxidation in BDA was delayed with alginate edible coating and accelerated with storage at higher temperatures and longer periods. This result correlates with the previous results described in Section 3.1. Therefore, the color changes in the CSA system have the potential to become an alternative tool for detecting the quality of food products.

#### 3.2.2. LDA

**Figure 4:**
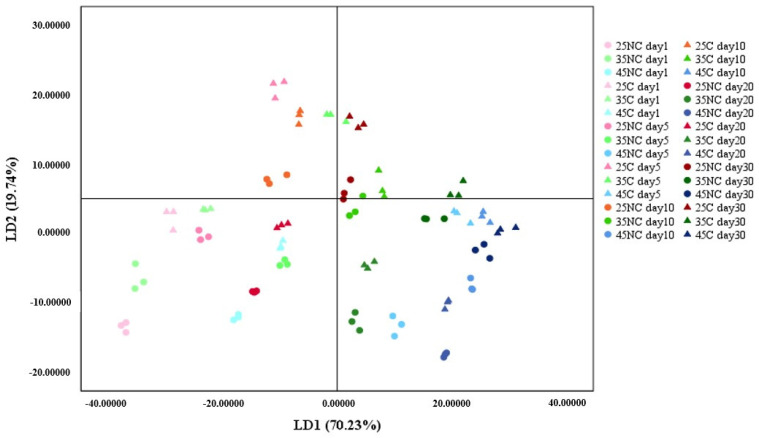
LDA analysis of boiled-dried anchovy over storage period.

The LDA method was used to investigate whether the oxidation level of the samples could be determined based on the results of the CSA system. As shown in Figure 4, the LDA score plot is composed of two different principal components accounting for 89.97% of the total variation (LD1 = 70.73% and LD2 = 19.24%). All within-samples were grouped successfully, and between-samples were classified successfully but with one overlap (25C day 30 and 35C day 5). The results were analyzed as a function of the presence of coating, storage temperature, and storage period.

The coated BDA samples are relatively positioned in the right upper segment compared to the non-coated BDA samples. For instance, 25C is placed in the right upper part compared to 25NC on day 1, 35C is in the right upper part compared to 35NC on day 10, and 45C is positioned in the right upper segment compared to 45NC on day 30. In addition, BDA stored at a lower temperature is positioned in the left upper segment compared to that stored at a higher temperature. For instance, both coated and non-coated BDA samples stored at 25 °C are positioned in the left upper segment compared to those stored at 35 °C for the same incubation period. A similar tendency can be found between both coated and non-coated samples stored at 35 °C and 45 °C. Furthermore, both coated and non-coated BDA samples shifted to the right plane as a function of a longer incubation period, except on day 20. As a result, compared to the samples stored for a day, the samples incubated for 30 days are positioned on the right plane based on the origin (0.00) of the LD1 axis.

As the degradation of BDA quality was induced by lipid oxidation, the BDA samples tend to be placed on the right lower plane on the LDA graph. Thus, the results of CSA data can be interpreted in combination with the LDA method. The combination of the CSA system and LDA method can, thus, be a powerful tool to determine the quality of BDA caused by lipid oxidation.

### 3.3. Correlation between Conventional Methods and CSA System

**Figure 5:**
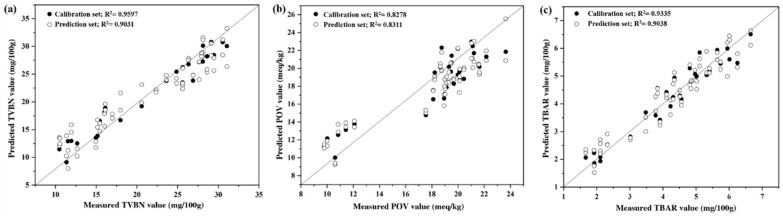
PLSR graph of correlation between CSA values and POV, (**a**); TVB-N, (**b**); TBA values, (**c**).

The PLSR method was used to determine the correlation between the conventional methods (TVB-N, POV, and TBA) and the CSA responses to the BDA samples. The results of PLSR prediction for the coefficient of determination ($r^{2}$), which indicates the accuracy and root mean square error that represents the precision following Equations (1) and (2), are presented in Figure 5. As described, the correlation coefficient between TVB-N and CSA data was $r_{c}{}^{2}$ = 0.960 and $r_{p}{}^{2}$ = 0.903, with RMSEC of 0.803 and RMSEP of 1.760; that of POV with CSA data was $r_{c}{}^{2}$ = 0.828 and $r_{p}{}^{2}$ = 0.831, with RMSEC of 0.931 and RMSEP of 1.303; and that of TBA with CSA data was $r_{c}{}^{2}$ = 0.934 and $r_{p}{}^{2}$ = 0.904, with RMSEC of 0.207 and RMSEP of 0.352. Therefore, an acceptable linear correlation between the CSA data and the TVB-N, POV, and TBARS results was found in the PSLR test results. These results indicate that the CSA system suggested in this study does not have significant differences from conventional methods for determining the quality of BDA samples, even though the samples in this study were coated with alginate film. In addition, following the previous study, the moderate value of the difference between RMSEP and RMSEC of the LDA model also provides a stable and reliable model for the prediction of the quality of BDA [33,36]. Therefore, the CSA system is an efficient alternative to conventional methods for detecting VOCs from fish products and determining food quality, regardless of surface modification.

## 4. Conclusions

This study aimed to prepare a novel CSA system as an alternative to traditional quality detection methods for surface-modified food products. To verify the efficacy of the prepared CSA system, its results were compared with those of conventional methods for evaluating lipid oxidation. TVB-N, POV, and TBARS tests were selected as conventional methods for determining the quality of BDA and were performed to evaluate the levels of lipid oxidation of BDA. Lipid oxidation of BDA was found to accelerate when the samples were stored at a higher temperature and had a longer incubation time. However, the alginate edible coating, which was selected as a surface-modifying agent in this study, was effective in delaying the lipid oxidation process in coated BDA samples. Overall, the colors of the sensor array in the CSA system changed significantly as a function of the presence of edible coating, storage temperature, and incubation period. It was thereby proven that the prepared CSA system could detect the quality degradation of BDA. In addition, the LDA method proved that the results from the CSA system could be successfully distinguished as per the lipid oxidation levels. Furthermore, to determine whether the CSA system could be applied as an alternative to conventional methods, the PLSR method was used to determine the correlation between the three dependent variables (TVB-N, POV, and TBA) and independent variables (CSA responses). As a result, a high correlation and good accuracy were obtained, which indicates that the lipid oxidation level of BDA can be detected through the CSA system. In conclusion, the CSA system could be used to detect the quality of surface-modified food products and the proposed CSA system in this study has the potential to be utilized as an alternative to conventional methods to determine the quality of fish products.

## Figures and Tables

**Table 1:** The dyes on PVDF membrane (3 × 3 cm^2^) for colorimetric sensor array.

Row/Column	Column 1	Column 2	Column 3
Row 1	Alizarin (No. 1)	Methyl red (No. 2)	Metanil yellow (No. 3)
Row 2	Bromocresol purple (No. 4)	Neutral red (No. 5)	Bromoxylenol blue (No. 6)
Row 3	Thymol blue (No. 7)	Bromocresol green (No. 8)	Cresol red (No. 9)

## Data Availability

All data generated or analyzed during this study are included in this published article.

## Supplementary Materials

The following supporting information can be downloaded at: https://www.mdpi.com/article/10.3390/foods12030638/s1. Table S1: Delta E valu.

## Abbreviations

Colorimetric sensor array (CSA), boiled-dried anchovy (BDA), linear discriminant analysis (LDA), partial least square regression (PLSR), gas chromatography (GC), liquid chromatography (LC), thiobarbituric acid-reactive substance (TBARS), total volatile basic nitrogen (TVB-N), peroxide value (POV), polyvinylidene fluoride (PVDF), trichloroacetic acid (TCA), butylated hydroxytoluene (BHT), thiobarbituric acid (TBA), deionized (DI), potassium iodide (KI), malondialdehyde (MDA), one-way analysis of variance (ANOVA), correlation coefficient ($r^{2}$), root mean square error (RMSE), total color difference (∆*E*), volatile organic components (VOCs), red, green, blue (RGB).
