# Supplementary material for: Preparation of Colorimetric Sensor Array System to Evaluate the Effects of Alginate Edible Coating on Boiled-Dried Anchovy
Source: Foods. 2023 Feb 2;12(3):638. doi: 10.3390/foods12030638 (PMC9913907; doi:10.3390/foods12030638)
Supplement: Supplementary file 1 [file foods-12-00638-s001.zip › foods-2099139-supplementary.pdf]

Table S1. Delta E value.

Sample/ Dye No.	1	2	3	4	5	6	7	8	9
day 1 25NC	30.84	33.91	23.07	43.53	19.61	60.41	101.98	110.48	49.09
day 1 25C	2.99	12.95	26.12	12.80	31.46	18.53	136.51	41.87	5.81
day1 35NC	41.96	28.25	48.89	33.50	20.25	25.21	136.71	95.48	24.44
day1 35C	10.87	45.09	22.56	2.41	12.23	24.59	131.47	76.78	43.70
day1 45NC	56.43	42.17	60.67	53.88	60.38	20.39	97.70	110.15	8.08
day1 45C	29.04	18.06	52.21	11.05	42.78	21.41	133.16	93.34	4.16
day5 25NC	37.39	11.86	18.53	4.16	48.28	29.63	136.48	85.26	22.76
day5 25C	7.93	12.77	6.50	30.21	47.53	38.17	132.51	65.62	34.47
day5 35NC	69.45	27.69	32.77	17.60	35.77	25.86	118.39	107.72	0.00
day5 35C	21.28	52.89	47.36	16.15	52.81	26.83	130.97	51.49	60.85
day5 45NC	61.42	19.54	28.60	20.92	77.51	37.28	131.55	78.64	30.52
day5 45C	53.43	30.05	9.76	36.60	66.46	41.13	130.85	61.28	33.70
day10 25NC	50.13	6.16	22.84	27.02	44.38	29.77	133.12	71.23	24.08
day10 25C	26.89	35.70	34.83	30.61	30.84	24.96	134.97	49.94	36.99
day10 35NC	71.33	17.60	37.57	11.84	56.46	21.61	136.39	42.51	38.57
day10 35C	55.19	22.14	29.25	25.14	56.75	31.38	133.52	57.29	40.02
day10 45NC	79.32	26.40	33.10	67.76	63.91	39.06	121.66	72.15	40.23
day10 45C	64.97	7.58	28.01	49.33	72.96	40.61	125.13	50.94	42.22
day20 25NC	79.73	26.47	34.53	29.68	27.99	23.96	123.99	104.78	15.81
day20 25C	69.56	16.38	43.46	5.54	36.51	26.42	129.14	102.76	36.42
day20 35NC	79.46	8.34	31.29	31.51	46.82	32.14	124.99	102.81	6.35
day20 35C	63.13	28.84	49.52	37.36	69.14	29.20	133.09	65.26	44.56
day20 45NC	66.36	26.41	35.79	31.43	108.76	12.65	136.48	91.30	19.84
day20 45C	64.35	12.00	28.99	28.92	78.18	38.36	135.75	70.56	24.50
day30 25NC	66.45	37.35	31.26	3.35	67.06	16.70	127.12	83.16	24.38
day30 25C	51.15	29.06	35.71	29.53	72.13	19.90	96.02	50.91	30.12
day30 35NC	52.97	41.70	9.71	17.87	73.95	35.63	135.08	55.73	50.11
day30 35C	53.36	39.02	35.11	31.23	73.45	26.76	129.60	52.74	53.84
day30 45NC	67.02	37.52	37.49	40.16	88.23	40.03	136.55	74.25	55.97
day30 45C	57.40	24.35	37.18	41.56	74.73	31.31	129.53	66.53	39.11
Average	51.39	25.94	32.42	27.42	55.24	29.66	128.01	74.77	31.36
Order	4	9	5	8	3	7	1	2	6
